# Effect of an herbal/botanical supplement on recovery from delayed onset muscle soreness: a randomized placebo-controlled trial

**DOI:** 10.1186/1550-2783-11-27

**Published:** 2014-06-13

**Authors:** Corey A Rynders, Judy Y Weltman, Sara D Rynders, James Patrie, John McKnight, Frank I Katch, Jay Hertel, Arthur Weltman

**Affiliations:** 1 Department of Kinesiology Exercise Physiology Core Laboratory, University of Virginia, Charlottesville, Virginia, USA; 2 Department of Human Movement Sciences, Human Performance Laboratory, Old Dominion University, Norfolk, VA, USA; 3 Jordan Young Institute, Virginia Beach, VA, USA

**Keywords:** DOMS, Blue-green algae, Eccentric exercise

## Abstract

**Background:**

We examined the effects of a proprietary herbal/botanical supplement (StemSport, Stemtech, San Clemente, CA.) suggested to increase circulating stem cells, decrease inflammation, and attenuate exercise induced muscle damage on recovery from delayed onset muscle soreness (DOMS).

**Methods:**

Sixteen subjects (male = 7, female = 9; age 23.8 ± 10 years; height 171.9 ± 10 cm, mass 72.2 ± 15 kg) were randomized in a crossover, double-blind, placebo controlled trial to receive a placebo or StemSport supplement (6150 mg/day) for 14 days. DOMS was induced on day 7 for both placebo and active conditions in the non-dominant elbow flexor group with repeated eccentric repetitions. Muscle swelling (biceps girth), elbow flexor isometric strength (hand held dynamometer), muscle pain/tenderness (visual analog scale), range of motion (active elbow flexion and extension), and inflammation (hsCRP, IL6, and TNF-α) were measured at baseline and at 24 h, 48 h, 72 h, and 168 h (1 week) post eccentric exercise. The crossover washout period was ≥14 days.

**Results:**

No significant condition-by-time interactions between placebo and StemSport supplementation were observed with regard to measures of pain (p = 0.59), tenderness (p = 0.71), isometric strength (p = 0.32), elbow flexion (p = 0.45), muscle swelling (p = 0.90), or inflammation (p > 0.90). Decrements in elbow extension range of motion 48 h post-exercise were less after StemSport supplementation (Δ elbow extension 48 h post; StemSport, −2.0 deg; placebo, −10 deg; p = 0.003).

**Conclusions:**

These data suggest that compared to placebo, StemSport supplementation does not improve outcome measures related to muscle recovery after acute upper-arm induced DOMS.

## Background

Delayed onset muscle soreness (DOMS) occurs following a bout of unaccustomed exercise in both novice and experienced athletes. DOMS is associated with muscle pain, decreased range of motion, muscle fiber disruption, altered joint kinematics, decreased strength, and acute tissue damage; each of which contribute to an impairment in future athletic performance and/or predispose individuals to injury [1,2]. Activities that involve high force eccentric muscle loading (e.g. plyometric exercises, the lowering phase of resistance training, and downhill running) induce the most severe cases of muscle damage. Symptoms of diffuse pain and tenderness associated with DOMS typically subside within 5 to 7 days after the inciting event. As such, studies evaluating the time course of DOMS typically include post-exercise data collection intervals as long as 168-h (1-wk) into the recovery period.

Delayed onset muscle soreness is a multi-factorial process and potential mechanistic theories include both anatomical/physiological and biochemical components. For example, anatomical/physiological mechanisms include connective tissue damage and muscular micro-trauma, and biochemical mechanisms include inflammation, and oxidative stress. Acute elevations in perceived pain and tenderness are the result of nociceptor stimulation in damaged muscle fibers and surrounding connective tissue [3]. Chronic symptoms of pain and tenderness are likely due to increased intramuscular pressure from the local pro-inflammatory response (e.g. IL-1β, hsIL-6, TNF-α, hsCRP, and others) which peaks in the early phase of recovery and typically persists for 5–7 days after eccentric exercise [3-5].

Therapeutic modalities for the management of DOMS related symptoms are numerous and include cryotherapy, stretching, massage, compression, ultrasound, oral non-steroidal anti-inflammatory drugs (NSAIDS), and exercise [2]. In addition, several dietary supplements have been tested (e.g. protein powders, vitamin C, fish oil, and chondroitin sulfate) with varying success (see review by Connolly et al. [6]). The present placebo-controlled study examined the effects of a proprietary supplement, StemSport (StemSport, Stemtech, San Clemente, CA.), on the severity and time course of DOMS following acute eccentric upper arm exercise. StemSport contains an extract from the botanical Aphanizomenon flos-aquae (AFA; blue-green algae), which has recently been shown to elevate human bone marrow derived stem cells [7]. Jensen et al. reported that a novel compound from AFA binds to the ligand-binding area of human L-selectin. L-selectin appears to play a role in cell adhesion and the release of bone marrow stem cells into the circulation [7]. Drapeau et al. recently hypothesized that bone marrow-derived stem cells may accelerate the tissue regeneration process in some animal models of injury and may play a role in recovery from muscle damaging exercise [8].

StemSport also contains a proprietary blend of herbal antioxidants, and anti-inflammatory substances (Table 1). Preliminary data suggest that supplementation with StemSport may accelerate tissue repair and restore muscle function earlier than would occur otherwise [7]. The manufacturer of StemSport claims that “by assisting in increasing the number of adult stem cells in the bloodstream the StemSport concept may help your body naturally repair, rebuild and recover faster, so you can return to activity and athletic participation more quickly” [9].

**Table 1:** StemSport ingredient list and purported benefits

**Ingredient**	**Amount per serving**	**Purported benefit**
**1. Aphanizomenon flos-aquae extract**	1000 mg	Increase the number of circulating stem cells; muscle repair [7,8]
2. Proprietary Herbal/Botanical Blend*	1575 mg	
Cats Claw	–	Antioxidant [16]
Mangosteen	–	Antioxidant [17]
Rehmannia	–	Anti-inflammatory [18]
Berry extracts	–	Antioxidant
Nattokinase	–	Anti-inflammatory/fibrinolytic [19,20]
Serrapeptase	–	Anti-inflammatory/fibrinolytic [20]
Curcumin	–	Antioxidant/anti-inflammatory [21,22]

*Specific doses not provided by the manufacturer.

Many commercially available supplements are often promoted without conclusive research demonstrating their efficacy. This present randomized, placebo-controlled, cross-over study examined the effects of StemSport supplementation on the inflammatory response, muscle function, and perceptions of pain and tenderness associated with upper arm delayed onset muscle soreness (DOMS). We hypothesized that compared to placebo, StemSport would accelerate the rate of DOMS recovery.

## Methods

### Subjects

Subjects were healthy males (n = 7) and females (n = 9) between the ages 20 and 38 years. Subjects were of normal weight (mean ± SD, Mass = 72.2 ± 14 kg; Body Fat = 24.4 ± 5%) and not currently participating in a structured resistance or aerobic endurance training program (resistance exercise was performed ≤ 30 min/day, 1 day/week and low to moderate aerobic exercise was performed ≤ 30 min/day, 3 days/week; subjects were asked to refrain from performing high intensity exercise resistance/aerobic training for the duration of the study). Exclusion criteria included a history of vascular disease, pregnancy, recent injury or surgery to their non-dominant elbow or upper arm musculature, soft tissue compression complications such as open wounds/broken skin of the non-dominant shoulder, arm, wrist or hand, neurological impairments, such as paresthesia or peripheral neuropathies, circulatory insufficiencies, such as diabetes, hemopheila, thrombophlebitis or phlebothrombosis, and regular use of over-the-counter and prescription pain and inflammation medications, or any agent known to treat the pain and tenderness associated with DOMS. The Institutional Review Board at the University of Virginia approved the study and subjects provided written informed consent prior to participation.

### Design

**Figure 1:**
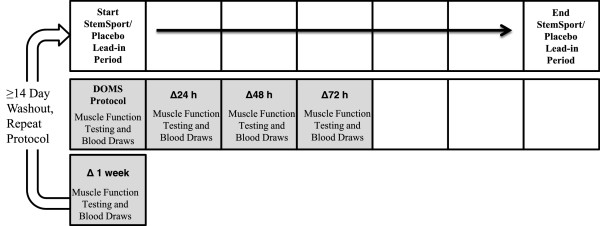
**Study timeline.** Subjects were administered either active or placebo for a 7 day lead in period. After the lead-in period, baseline measures of muscle function were assessed. Subjects then performed a standardized DOMS protocol for the upper arm. Stemsport/placebo supplementation continued for 7 days post-DOMS. Muscle function outcome measures were repeated for 3 consecutive days after the DOMS protocol and once again 7 days after the DOMS protocol. Subjects repeated the protocol (opposite condition) after a minimum 14-day washout period.

A study timeline is provided in Figure 1. Subjects were initially examined by the study physician in the General Clinical Research Center (GCRC) at UVA to ensure pre-screening eligibility. Eligible subjects were given a 14 day supply of StemSport or a placebo. Subjects and members of the study team were blinded to the treatment condition. After 7-days of lead-in supplementation, subjects returned to the GCRC to complete baseline tests of upper arm swelling, range of motion, and visual analog scales to evaluate perceptions of elbow flexor pain and tenderness. Blood samples were obtained for analysis of highly sensitive C-reactive protein (hsCRP), tissue necrosis factor-alpha (TNF-α), and interleukin-6 (IL-6). After baseline testing, subjects performed an upper-arm DOMS exercise protocol. Tests of upper arm swelling, range of motion, pain and tenderness visual analog scales, and blood draws were repeated 24 h, 48 h, 72 h, and 168 h (1 week) after the DOMS exercise protocol in each condition (StemSport/Placebo).

### StemSport and placebo supplementation

The StemSport ingredient list is presented in Table 1. Subjects were instructed to adhere to the following daily dosing schedule according to manufacturer recommendations: 1000 mg of Aphanizomenon flos-aquae extract 3 times per day in conjunction with food (breakfast, lunch, and dinner) and 1575 mg of a proprietary herbal/botanical blend twice per day in conjunction with food (breakfast and dinner). Prior to the DOMS protocol subjects ingested an extra 1000 mg dose of Aphanizomenon flos-aquae and an extra 1575 mg dose of the herbal/botanical blend. The extra dose was ingested with water at least 1-hour prior as per manufacturer instructions. No food was ingested because the pre-DOMS blood samples were collected in the fasted state. The placebo was visually similar to StemSport but consisted of a biologically inactive substance (1000 mg of encapsulated corn starch). The time of day that the supplements were taken was standardized to meal time (e.g. breakfast, lunch, and dinner). Subjects were required to maintain a pill diary throughout the study and were instructed to forfeit any capsules not ingested during the study period. Over-the-counter analgesic and anti-inflammatory medications (i.e. Tylenol, Advil, Ibuprofen, Motrin, Bextra, Celebrex, etc.) were prohibited during the supplementation period.

An independent manufacturer (StemSport, Stemtech, San Clemente, CA.) packaged and the supplements/placebo. Supplements (placebo/active) were stored and distributed to subjects by the University Investigational Pharmacy. None of the members of the study team (except the pharmacist) knew the identity of the supplements during the study. The order of supplement consumption (placebo or active) was randomly assigned based on a code known only to the pharmacist and the study biostatistician.

### Pain and tenderness

A pressure algometer (Wagner Instruments, Greenwich, CT) was used to assess the pressure sensitivity and pain tolerance of the soft-tissue 5 cm proximal to the elbow joint line of the biceps brachii muscle. Each subject received 0.91 kg of compression and recorded their perceived level of pain on a visual analog scale (VAS) from 0–10, 0 indicating no pain and 10 representing the worst pain ever experienced. Perceived tenderness of the biceps brachii was also assessed using the same visual analog scale. A standard plastic measurement tape with 1 mm gradations was used to measure the girth of the non-dominant arm 5 cm proximal to the elbow joint line.

### Biceps peak force

Isomeric elbow flex strength of the dominant arm was measured at an angle of 90 degrees using a hand-held dynamometer (Hoggan Health Industries, West Jordan, Utah). The test was performed in the standing position with the subject’s upper arm resting against a wall to ensure exclusive contraction of the elbow flexors.

### Range of motion

A standard goniometer (Model G300, Whitehall Manufacturing, City of Industry, CA) was used to measure the degrees of active elbow range of motion (extension and flexion).

### Inflammatory assays

Subjects were fasted at least 6 hours prior to the blood draws at each time point. They were not allowed to consume any food and/or drink prior to the other baseline measurements or DOMS protocol. Water consumption was allowed, but the intake volume was not measured. TNF-alpha and IL-6 concentrations were measured in serum using high-sensitivity ELISA assays. The assay sensitivities were 0.5 pg · ml − 1 for TNF-α and 0.3 ng · ml − 1 for IL-6; the mean intra- and interassay coefficients of variation were 6.7% and 13.4% for TNF-α, and 7.4% and 7.8% for hsIL-6. CRP concentrations were measured by a chemiluminescent assay (Diagnostic Products Corporation, Immulite 2000, Los Angeles, CA), the assay sensitivity was 0.1 mg · l − 1 and the mean intra- and interassay coefficients of variation were 6.7%.

### DOMS

After completion of the baseline tests above, subjects performed an upper arm DOMS protocol as described by Denegar et al. [10]. Proper positioning, timing, and form for the DOMs protocol were thoroughly explained by the study team and subjects were allowed to practice the protocol with light weights prior to the first supplementation period/actual day of testing. DOMS protocol prior to actually performing it. A preacher curl bench with adjustable height was used to isolate the biceps brachii muscle group of the non-dominant arm. Subjects repetitively performed all eccentric contractions, while study personnel performed the concentric phase of the bicep curl. The DOMS protocol was designed to be performed with continuous repetitions until exhaustion (i.e. there was not a prescription of sets and repetitions and there was no allotted rest interval within the protocol). Each subject started with a 15.91 kg dumbbell and performed eccentric contractions until unable to lower the weight under control over a three second count (if unable to perform one successful repetition with a 15.91 kg weight, subjects began with a 13.63 kg weight). The weight decreased in 2.27 kg (5 lbs) increments after a participant could no longer complete repetitions at a given weight all the way down to a final weight of 2.27 kg (5 lbs). The DOMS protocol was complete once the subject was unable to lower a 2.27 kg weight under control. Verbal cues were provided throughout the fatigue protocol, including encouragement to exert full strength and reminders about the minimum three second count. Upon completion of the DOMS protocol, each subject was provided with an arm sling to secure the non-dominant arm against the body with the elbow flexed at 90°. Subjects were asked to wear the sling up to the start of day 3 (72-hours post-DOMS exercise) and remove it only to perform activities of daily living (i.e. bathing, getting dressed, sleeping, driving).

### Follow-up measures

Measures of pain and tenderness, muscle function, and blood draws for inflammatory markers were repeated 24-hours, 48-hours, 72-hours, and 168-hours (1-week) following DOMS protocol. After the 1-week post-exercise visit, subjects completed a 14-day washout period and then repeated the protocol exactly as outlined above with opposite treatment condition (StemSport or Placebo). Subjects were asked to maintain similar dietary patterns throughout the duration of the study.

### Statistical analyses

Separate RM-ANOVA models were used to evaluate the effects of StemSport versus placebo on the primary outcomes. The primary outcome measures were change in perceived pain and tenderness (VAS scales), change in edema (girth), change in muscle function (range of motion and biceps peak force), and change in inflammation (hsCRP, TNF-alpha, and IL-6) 24-hours, 48-hours, 72-hours, and 168-hours post-DOMS. Treatment status (StemSport or placebo) was the between group factor and time was the within group factor. Baseline (pre-DOMS) values were used as covariates. Comparisons between conditions were tested by way of a Bonferoni adjusted linear contrast of means. A p ≤ 0.05 decision rule was utilized as the null hypothesis rejection criterion for the individual adjusted statistical tests. SAS version 9.2 (SAS Institute Inc, Cary, NC, USA) was used to conduct the data analyses.

## Results

### Safety

There were no serious adverse events during the study period. Subjects reported unusual urine oder (n = 1), tiredness (n = 1), dry mouth (n = 1), headaches (n = 2), and nausea (n = 1) while on StemSport supplementation and tiredness/headaches (n = 1) while on the placebo. There were no subject dropouts.

### Pain and tenderness

**Figure 2:**
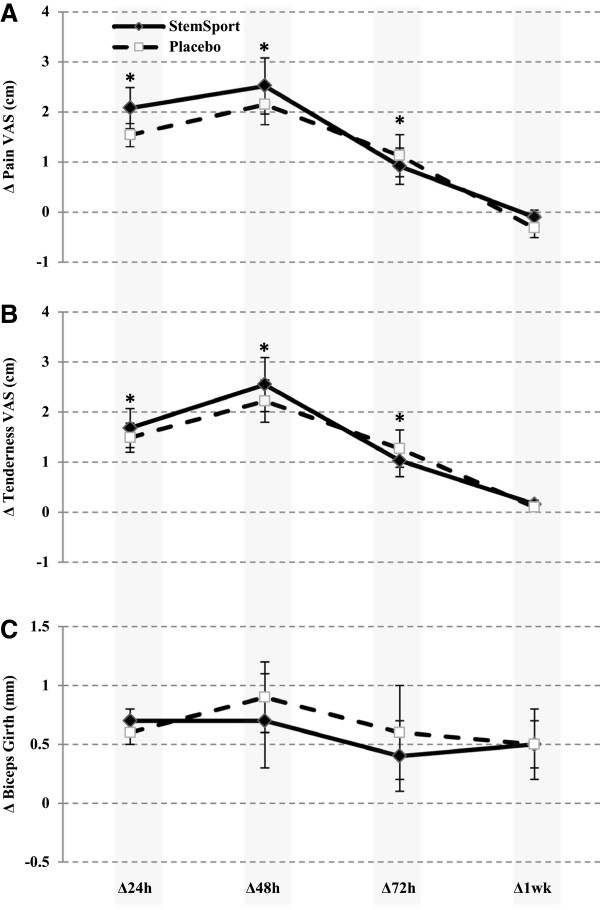
**Baseline adjusted comparison of the mean change (±SEM) in (A) elbow flexor pain and (B) tenderness, and (C) biceps girth between StemSport and placebo at 24, 48, 72 and 168 hours post-DOMS exercise.** *Perceived ratings of muscle pain and tenderness were significantly increased in both conditions for 72 hours post-exercise (p < 0.001; **A** and **B**).

Perceived ratings of muscle pain and tenderness were significantly increased in both conditions for 72 hours post-exercise (p < 0.001; Figure 2A and B). There were no differences in pain or tenderness ratings between conditions at any time point (baseline adjusted comparison of the mean change in pain and tenderness at 24, 48, 72, and 168 hours post-exercise, p = 0.99). Biceps girth, a measure of local tissue swelling, was increased for 48-hours post-exercise in both conditions (p < 0.03; Figure 2C).

### Measures of muscle function

**Figure 3:**
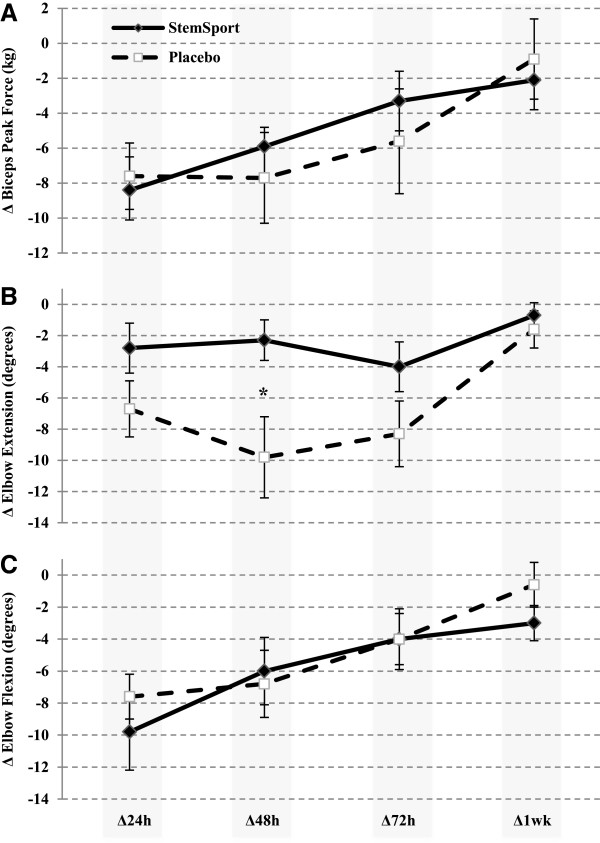
**Baseline adjusted comparison of the mean change (±SEM) in (A) biceps peak force, (B) elbow extension range of motion, and (C) elbow flexion range of motion between StemSport and placebo at 24, 48, 72 and 168 hours post-DOMS exercise.** *p = 0.003, significantly different from placebo. For biceps peak force, 0.91 kg equates to 2 pounds or 8.9 Newtons.

Biceps peak force was decreased for 72 hours in both the placebo (p < 0.02; Figure 3A) and StemSport condition (p < 0.05; Figure 3A). Significant decrements in elbow extension range of motion were observed for 72 hours during the placebo (p < 0.001; Figure 3B), and range of motion tended to be reduced during StemSport supplementation (p < 0.14; Figure 3B). Elbow flexion range of motion was significantly reduced in both groups for 72 hours (p < 0.03; Figure 3C). The only significant difference in muscle function between conditions was elbow extension range of motion (placebo, 10 degree decrement in elbow extension range of motion at 48 hours post-exercise versus StemSport, 2 degree decrement in elbow extension range of motion; p = 0.003; Figure 3B). Overall, less extension range of motion decrement post-exercise was found with supplementation of StemSport versus the placebo up to 72-hrs post exercise. All measures of muscle function returned to baseline values 1 week post-exercise (p > 0.07; Figure 3A-C).

### Inflammatory markers

**Figure 4:**
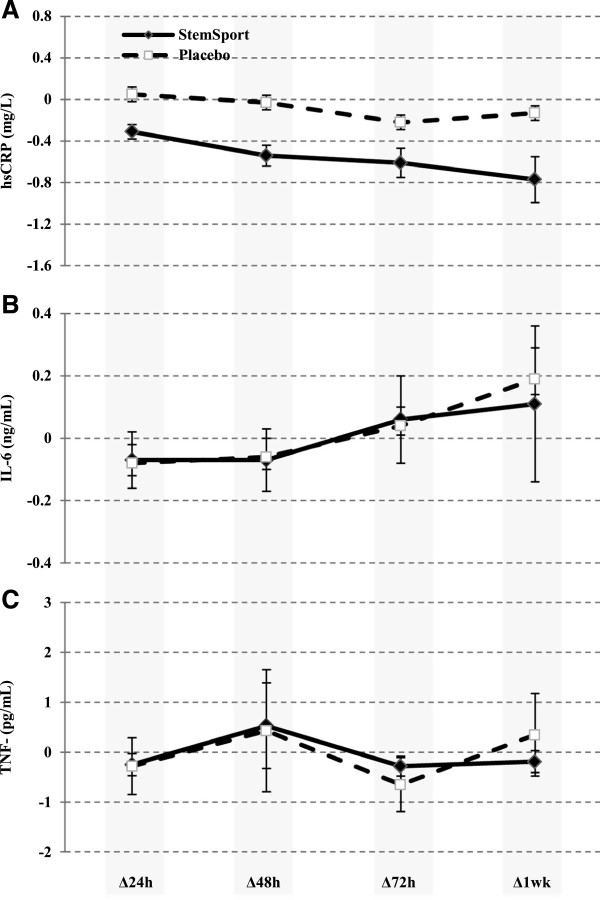
Baseline adjusted comparison of the mean change (±SEM) in (A) hsCRP, (B) IL-6, and (C) TNF-α between StemSport and placebo at 24, 48, 72 and 168 hours post-DOMS exercise.

Complete blood work was completed on 14 of 16 subjects and the data are presented in Figure 4A-C (missing data due to difficulties with blood draws and laboratory analysis). Systemic markers of inflammation did not significantly change from baseline values in either condition (hsCRP, p-value for time = 0.24; IL-6, p-value for time = 0.05; TNF-α, p-value for time = 0.24). There were no differences between groups for plasma markers of inflammation (p = 0.90).

## Discussion

The main finding of the present study is that StemSport did not accelerate recovery from an acute bout of single upper-arm eccentric exercise in non-resistance trained adults.

StemSport contains the fresh water blue-green algae, AFA, which has been studied primarily for its antioxidant/anti-inflammatory properties [11]. The effects of AFA on inflammation are limited to animal studies [11]. To our knowledge, the present study is the first to examine the effects of AFA on systemic inflammation and other markers of DOMS in humans.

Most recently, AFA has been suggested to be a potential bone marrow stem cell mobilizer [7]. Studies from Jensen et al. (2007) and Drapeau et al. (2010) indicate that a novel compound from AFA appears to play a role in the release of bone marrow stem cells into the circulation, and it has been suggested that bone marrow-derived stem cells may accelerate the tissue regeneration process in some animal models of injury [7,8]. It has been further hypothesized that AFA plays a role in recovery from muscle damaging exercise via increasing bone marrow-derived stem cells, although this has not been tested directly in humans [8].

In a placebo-controlled double-blind crossover study, a 5:1 concentrate of AFA concentrate fed to healthy volunteers (n = 12) produced a 25 ± 1% increase in number of circulating CD34+ stem cells at 60 minutes (p < 0.0001) [7]. In contrast, the placebo only produced minor fluctuations in levels of stem cells in the blood circulation over 2 hours. It has been hypothesized that acute increases in post-exercise circulating levels of stem cells may be beneficial for tissue regeneration and recovery [8]. Stem cell counts (e.g. CD34+) were not specifically measured in the present study, however, given that recovery of muscle function was similar between conditions, it is unlikely that any AFA induced change in circulating stem cells plays a major role in recovery from upper arm DOMS.

In agreement with previous studies in the literature, we did not observe an association between circulating inflammatory markers and others markers of DOMS (e.g. pain and tenderness) [12,13]. However, this may be related to the relatively small muscle mass utilized in our DOMS protocol which may not have been a potent stimulus for increasing circulating cytokines. Future studies should attempt to study AFA supplementation in response to an eccentric exercise protocol designed to produce a much larger inflammatory response (i.e. downhill running).

Leukocytes, neutrophils, and monocytes/macrophages are attracted to damaged tissue within hours of tissue injury and remain present for up to 24 hours, or as has been shown in macrophages, up to 14 days [14]. Neutrophils and macrophages assist in degradation of damaged muscle tissue primarily through production of reactive oxygen and nitrogen species (RONS). Degradation of damaged tissue is also initiated by the expression of many local pro- and anti-inflammatory cytokines (e.g. IL-6, TNF-α, IL-1β, etc.). Circulating IL-6, which has both pro- and anti-inflammatory functions, is related to the level of DOMS, and there is some debate as to whether the post-exercise IL-6 response is required for muscle adaptation [5]. Elevated levels of IL-6 persist for at least 48 hours after eccentric upper arm exercise [15]. Less is known about the post-exercise time course of TNF-α, although studies have detected elevated levels of TNF-α for up to 5 days during DOMS [15]. The present data do not support a role of AFA in suppressing circulating levels of IL-6, TNF-α, or CRP in humans in the basal state or in response to an acute bout of upper arm eccentric exercise designed to induce DOMS.

Besides AFA, StemSport contains a proprietary blend of several herbal substances potential antioxidant or anti-inflammatory properties (Cat’s Claw [16], Mangosteen juice [17], Radix Rehmanniae Preparata [18], Nattokinase [19,20], Serrapeptase and [20], and Curcumin [21]; see Table 1). For example, Curcumin, an ingredient derived from the spice Tumeric, has been shown in a few studies to reduce DOMS related pain and swelling [17,22] and has a potential role is reducing obesity-related inflammation. However, our data tend to agree with the majority of studies in the literature which show that oral antioxidant supplementation has minimal to no effect on reducing subjective ratings of pain, tissue swelling, or decrements in muscle function after a bout of eccentric exercise [2,23-25]. It should be noted that data in the literature now support an inhibitory effect of oral antioxidant supplementation on the skeletal muscle adaptations exercise [26]. In addition, supplementation with the popular antioxidant ascorbic acid has been shown to delay the recovery process [24].

A possible limitation of this study was the use of DOMS to examine the utility of StemSport. It is possible that the amount of tissue damage associated with the DOMS protocol may have been too great for StemSport to have an effect. It is possible that if a less disruptive regimen was applied (e.g. strength training) StemSport supplementation may enhance chronic adaptations to whole body resistance training. Also, future studies may consider investigating the effects of AFA, independent or in combination with the other herbal substances. Strengths include study design (placebo-controlled, double-blinded, randomized trial) and measurement of both subjective markers of DOMS as well as bio-markers of the inflammatory process.

## Conclusions

Supplementation with StemSport compared to a placebo was unable to accelerate recovery from upper arm eccentric exercise. In agreement with the majority of studies in the literature, dietary supplementation with antioxidant/anti-inflammatory substances likely provides minimal to no benefit for reducing the acute symptoms associated with delayed onset muscle soreness.

## Competing interests

The authors declare that they have no competing interests. The study was funded in part by an urestricted gift to the Curry School of Education Exercise Physiology Fund from StemTech International, Inc. San Clemente, CA. FIK, JH, and AW served as scientific consultants for StemTech International.

## Authors’ contributions

CAR, JH, FIK, and AW contributed to the study conception and design, SDR and JM screened the subjects and provided medical oversight, CAR, JYW acquired the data, JP performed the data analysis, CAR, JH, FIK, and AW interpreted the data; All authors were involved in drafting the manuscript and have given final approval of the published version.
